# Identity of the holotype and type locality of *Rhabdophis leonardi* (Wall, 1923) (Colubridae: Natricinae), with notes on the morphology and natural history of the species in southwestern China

**DOI:** 10.1002/ece3.10032

**Published:** 2023-05-02

**Authors:** Shi‐Jun Yang, Alan H. Savitzky, David J. Gower, V. Deepak, Akira Mori, Rahul Khot, Jing‐Song Shi, Li Ding, Mian Hou, Hai‐Yuan Xu, Qin Wang, Guang‐Xiang Zhu

**Affiliations:** ^1^ College of Life Science Sichuan Agricultural University Ya'an China; ^2^ Department of Biology Utah State University Logan Utah USA; ^3^ Natural History Museum London UK; ^4^ Senckenberg Natural History Collections Dresden Germany; ^5^ Department of Zoology, Graduate School of Science Kyoto University Sakyo Kyoto Japan; ^6^ Bombay Natural History Society Fort, Mumbai India; ^7^ Key Laboratory of Vertebrate Evolution and Human Origins of Chinese Academy of Sciences Institute of Vertebrate Paleontology and Paleoanthropology Chinese Academy of Science Beijing China; ^8^ Chengdu Institute of Biology Chinese Academy of Sciences Chengdu China; ^9^ Sichuan Normal University Chengdu China

**Keywords:** distribution, morphology, Natricidae, Sinlum Kaba, snakes, systematics

## Abstract

The original description of *Natrix leonardi* (currently *Rhabdophis leonardi*) by Frank Wall in 1923, based on a specimen from the “Upper Burma Hills,” lacked important morphological details that have complicated the assignment of recently collected material. Furthermore, although the holotype was never lost, its location has been misreported in one important taxonomic reference, leading to further confusion. We report the correct repository of the holotype (Natural History Museum, London), together with its current catalog number. We also describe key features of that specimen that were omitted from the original description, and provide new details on the morphology of the species, including sexual dichromatism unusual for the genus, based upon specimens from southern Sichuan, China. *Rhabdophis leonardi* is distinguished from its congeners by the following characters: 15 or 17 DSR at midbody and 6 supralabials; distinct annulus around the neck, broad and red in males, and narrow and orange with a black border in females; dorsal ground color light green or olive; some lateral and dorsal scales possessing black edges, the frequency of black edges gradually increasing from anterior to posterior, forming irregular and ill‐defined transverse black bands; eye with prominent green iris; black ventral spots with a red edge, most numerous at midbody but extending halfway down the length of the tail. In southwestern China, this species is frequently found at 1730–2230 m elevation. It has been documented to prey upon anuran amphibians, including toads. A recently published phylogenetic analysis showed this species to be deeply nested with the genus *Rhabdophis*, as a member of the *R. nuchalis* Group. That analysis also revealed the existence of two closely related but geographically distinct subclades in the molecular analysis, one of which may represent an unnamed taxon.

## Graphical abstract

This manuscript has involved correcting a long history of misinformation concerning the identity of the holotype of *Rhabdophis leonardi*, the circumstances surrounding several collections of snakes from that region, the location and unusual colonial history of the type locality, and the relevance of these questions to the taxonomic history of the species.

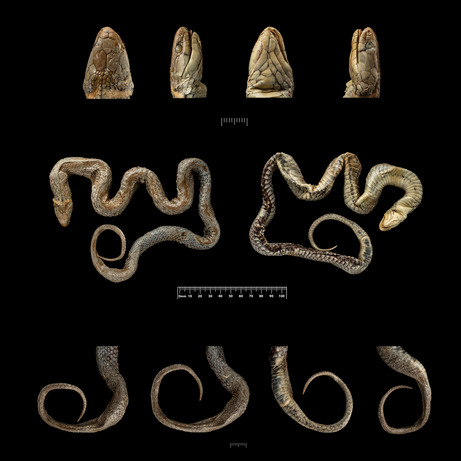

## 1 INTRODUCTION

The natricine genus *Rhabdophis* Fitzinger, 1843 is widely distributed across southern and eastern Asia, from northeastern India and Sri Lanka through China to Japan, and south to the islands of Malaysia and Indonesia. Note that the Natricinae, considered a subfamily here and by many authors, (e.g., Zheng & Wiens, 2016), is regarded as a family by other recent authors (e.g., Burbrink et al., 2020; Zaher et al., 2019). At either rank, the content and relationships of the clade are equivalent. Malnate (1960) assigned 15 species to *Rhabdophis* when he partitioned it from the expansive nominal genus *Natrix*. He characterized *Rhabdophis* as having a terrestrial habitus, with enlarged posterior maxillary teeth, usually following a diastema. He noted that many of the species reportedly possessed nuchal or nucho‐dorsal glands. Those integumentary glands had first been described in *Rhabdophis tigrinus* by Nakamura (1935) and soon thereafter were reported in other congeners by Smith (1938). Initially of unknown function, those glands were only known from *Rhabdophis*, some species of *Macropisthodon*, and the monotypic genus *Balanophis* Boulenger, 1893, erected by Smith to accommodate *Natrix ceylonicus* (Smith, 1938).

In the years since Malnate's resurrection of *Rhabdophis*, the genus has expanded to include 32 currently recognized species, several of them identified or included on the basis of molecular analyses. Formerly widespread species, such as the nominal *R. subminiatus* (the type species), *R. tigrinus*, and *R. nuchalis*, have been found to harbor cryptic diversity at the specific level (David & Vogel, 2021; Takeuchi et al., 2012; Zhu et al., 2022). Furthermore, a comprehensive analysis of the Asian natricines that possess nuchal or nucho‐dorsal glands, together with their nominal congeners, was conducted by Takeuchi et al. (2018), who determined that the genus *Macropisthodon*, as constituted at that time, was paraphyletic. Two of the species included in that study (*M. flaviceps*, the type species, and *M. plumbicolor*) were found to be nested among the species assigned to *Rhabdophis*, as was *Balanophis ceylonensis*, whereas *M. rudis* lay far outside the so‐called “nuchal gland clade” of Natricinae. Meanwhile, Figueroa et al. (2016) had found that *M. rhodomelas* also lies within *Rhabdophis*. Therefore, Takeuchi et al. (2018) formally synonymized *Balanophis* and *Macropisthodon* with *Rhabdophis*, uniting all species with nuchal or nucho‐dorsal glands within that single genus, together with a few populations that appear to have lost the integumentary glands secondarily. *Rhabdophis spilogaster* was recently sequenced, and it was moved to the genus *Tropidonophis* (Deepak et al., 2022), whereas the phylogenetic positions of *Rhabdophis auriculatus*, *R. chrysargus*, and *R. conspicillatus* are unclear at this time and remain to be resolved (Deepak et al., 2022). Of the 32 currently recognized species of *Rhabdophis*, 14 occur in China, including Taiwan: *R. adleri* Zhao, 1997; *R. chiwen* Chen, Ding, Chen and Piao, 2019; *R. confusus* David & Vogel, 2021; *R. formosanus* (Maki, 1931); *R. guangdongensis* Zhu et al., 2014; *R. helleri* (Schmidt, 1925); *R. himalayanus* (Günther, 1864); *R. lateralis* (Berthold, 1859); *R. leonardi* (Wall, 1923); *R. nigrocinctus* (Blyth, 1855); *R. nuchalis* (Boulenger, 1891); *R. pentasupralabialis* Jiang & Zhao, 1983; *R. siamensis* (Mell 1931); and *R. swinhonis* (Günther, 1868). A recent study by Zhu et al. (2022) revealed additional cryptic diversity within the derived worm‐eating clade of southwestern China, designated the *R. nuchalis* Group, to which *R. leonardi* belongs.

Meanwhile, the morphology and chemistry of the nuchal and nucho‐dorsal glands have been extensively studied and substantially clarified. The glands are now known to serve a defensive function by releasing noxious steroidal compounds known as bufadienolides that the snakes sequester from toxic prey (Hutchinson et al., 2007; Mori et al., 2012). Superimposing both the anatomical distribution of the integumentary glands (i.e., whether they occur along with the entire length of the body, as nucho‐dorsal glands, or are limited to the neck, as nuchal glands) and the dietary source of the toxins (whether from amphibians or insects) on the phylogeny, it is clear that *Rhabdophis* likely ancestrally possessed nucho‐dorsal glands that contain bufadienolides derived from a diet of anuran amphibians (Takeuchi et al., 2018; Yoshida et al., 2020). Importantly, the phylogeny also reveals that some members of a deeply nested clade that occurs in southwestern China and adjacent regions underwent a shift in their primary diet from frogs to earthworms. Accompanying that change in diet was a shift in the source of the sequestered defensive toxins from toads (Bufonidae) to lampyrid firefly larvae, both of which contain bufadienolide steroids (Yoshida et al., 2020).

In the course of identifying specimens belonging to the worm‐eating clade of *Rhabdophis* from Sichuan Province, China, we encountered inconsistencies in our comparison of the new specimens with the original description of *R. leonardi* (Wall, 1923). Furthermore, our attempts to compare our specimens to the holotype of that species were confounded by erroneous information on the repository of the holotype, a problem exacerbated at the time by travel restrictions and museum closings associated with the COVID‐19 global pandemic. Wall's (1923) original description of the holotype was insufficient to distinguish the species from among the greater diversity of *Rhabdophis* recognized today, and some critical attributes of the type specimen had been omitted, notably its sex. Ironically, this species appears to have the most extreme sexual dichromatism of any member of the genus. Finally, after the holotype had been located and our specimens had been determined to conform morphologically with *R. leonardi*, our efforts were further complicated by the presence of two well‐differentiated clades within that nominal species, as documented in the molecular assessment of the *R. nuchalis* Group by Zhu et al. (2022).

Here we correctly identify the repository of the holotype of *Natrix leonardi* Wall, 1923, describe the historical context behind the discovery of that specimen and the significance of the type locality, and provide important additional details on the morphology of the holotype, including its sex. We also address the phylogenetic relationships of species based on the recent work of Zhu et al. (2022) and a new molecular phylogenetic analysis. We describe in detail the morphology of a male specimen (for comparison with the female holotype; for the determination of sex of the holotype, see below), briefly describe several additional female specimens based on recent material, and describe a hatchling from the Sichuan population, including the coloration of these specimens in life and in preservative. We report on two additional localities in neighboring Yunnan Province, based upon field observations and photographs, and provide information on the natural history of the species in China. Finally, we discuss the likely association of the name *Rhabdophis leonardi* with one of the two molecular clades that currently bear that name and suggest fruitful directions for future studies.

## 2 MATERIALS AND METHODS

### 2.1 Phylogenetic analysis

A phylogenetic tree was inferred from analyses of concatenated sequences of one mitochondrial (*cyt b*) and one nuclear (*c‐mos*) gene. Sequences were aligned by Mega 5.0 (Tamura et al., 2011). A total of 1599 base pairs for 50 samples were analyzed in this study, and *Natriciteres olivacea* (Peters, 1854) was selected as an outgroup. The sequences used for constructing our phylogenetic tree are listed in Table 1. The phylogenetic analysis employed maximum likelihood (ML) and Bayesian inference (BI) methods. The best‐fitting model of sequence evolution for the BI analysis was determined using PartitionFinder 2.1.1 (Lanfear et al., 2017), separating all genes by codon position and identifying the best‐fitting partitions scheme, employing the Akaike information criterion (Akaike, 1974), and the phylogenetic analysis was performed using MrBayes 3.1.2 (Huelsenbeck & Ronquist, 2001). The parameters for the BI analysis included posterior distributions obtained by Markov Chain Monte Carlo (MCMC) analysis, with four chains for 10 million generations and sampled every 1000th generation. The first 25% of the trees were discarded (burn‐in). ML analyses were performed with RAxML 8.2.10 (Stamatakis, 2014) under the GTRGAMMA model, using 1000 bootstrap replicates.

**TABLE 1:** Information on sequences and their voucher specimens used in this study.

ID	Current genus and species name	Locality	Voucher specimen	Accession no. of GenBank		References
				*c‐Mos*	Cyt *b*	
1	*Natriciteres olivacea* (outgroup)	Democratic Republic of Congo	LSUMZ 41506	MT587299	MT587285	Deepak et al. (2021)
2	*Fowlea asperrimus*	Sri Lanka	HT0797	LC325346	LC325792	Takeuchi et al. (2018)
3	*Fowlea asperrimus2*	Sri Lanka	RS‐J	KC347480	KC347413	Pyron et al. (2013)
4	*Fowlea piscator*	Thailand	HT0347	LC325317	LC325763	Takeuchi et al. (2018)
5	*Fowlea piscator2*	Vietnam	HT0371	LC325318	LC325764	Takeuchi et al. (2018)
6	*Rhabdophis adleri*	Hainan, China	HT0831	LC325802	LC325356	Takeuchi et al. (2018)
7	*Rhabdophis auriculatus*	Mindanao, Philippines	FMNH275314	OK315944	/	Deepak et al. (2022)
8	*Rhabdophis callichromus*	Vietnam	HT0654	LC325770	LC325324	Takeuchi et al. (2018)
9	*Rhabdophis callichromus2*	Vietnam	HT0674	LC325771	LC325325	Takeuchi et al. (2018)
10	*Rhabdophis ceylonensis*	Sri Lanka	HT0787	LC325786	LC325340	Takeuchi et al. (2018)
11	*Rhabdophis ceylonensis2*	Sri Lanka	RS‐D	KC347384	KC347474	Pyron et al. (2013)
12	*Rhabdophis chiwen*	Jiguan Mountain, Sichuan, China	CIB116092	MN656331	MN656352	Piao et al. (2020)
13	*Rhabdophis chiwen2*	Jiguan Mountain, Sichuan, China	CIB116093	MN656332	MN656353	Piao et al. (2020)
14	*Rhabdophis chrysargos*	Malaysia	HT0342	LC325759	LC325313	Takeuchi et al. (2018)
15	*Rhabdophis chrysargos2*	Sabah, Malaysia	FMNH246185	OK315945	OK315834	Deepak et al. (2022)
16	*Rhabdophis conspicillatus*	Malaysia	HT0791	LC325788	LC325342	Takeuchi et al. (2018)
17	*Rhabdophis flaviceps*	Malaysia	HT0809	LC325801	LC325355	Takeuchi et al. (2018)
18	*Rhabdophis formosanus*	Taiwan, China	HT0033	LC325750	LC325304	Takeuchi et al. (2018)
19	*Rhabdophis guangdongensis*	Aizhai, Renhuay, Guangdong, China	SYS r000018	KF800920	KF800930	Zhu et al. (2014)
20	*Rhabdophis himalayanus*	Kachin State, Myanmar	CAS224420	KF800919	KF800929	Zhu et al. (2014)
21	*Rhabdophis lateralis*	Huangshan, Anhui, China	GP1195	KF765390	KF765395	Zhu et al. (2014)
22	*Rhabdophis lateralis2*	Tianshui, Gansu, China	SCUM090004	KF765391	KF765396	Zhu et al. (2014)
23	*Rhabdophis lateralis3*	Longnan, Gansu, China	SCUM090001	KF765392	KF765397	Zhu et al. (2014)
24	*Rhabdophis lateralis4*	China	GP613	GQ281785	JQ687444	Guo et al. (2012)
25	*Rhabdophis leonardi* (Clade B in Zhu et al. (2022))	Huili, Sichuan, China	SICAU201705026	MK987078	MK987071	Zhu et al. (2022)
26	*Rhabdophis leonardi* (Clade B in Zhu et al. (2022))	Panzhihua, Sichuan, China	SICAU201807001	MK987077	MK987070	Zhu et al. (2022)
27	*Rhabdophis leonardi* (Clade B in Zhu et al. (2022))	Panzhihua, Sichuan, China	SICAU201705027	MK987079	MK987072	Zhu et al. (2022)
28	*Rhabdophis leonardi* (Clade B in Zhu et al. (2022))	Huili, Sichuan, China	SICAU201705031	MK987080	MK987073	Zhu et al. (2022)
29	*Rhabdophis leonardi* (Clade B in Zhu et al. (2022))	Panzhihua, Sichuan, China	SCUM090009	KF800923	KF800933	Zhu et al. (2014)
30	*Rhabdophis leonardi* (Clade B in Zhu et al. (2022))	Daili, Yunnan, China	RDQ200905367	KF800922	KF800932	Zhu et al. (2014)
31	*Rhabdophis leonardi* (Clade C in Zhu et al. (2022))	Cayu, Xizang, China	SICAU201706023	MK987082	MK987075	Zhu et al. (2022)
32	*Rhabdophis leonardi* (Clade C in Zhu et al. (2022))	Cayu, Xizang, China	SICAU201508001	MK987081	MK987074	Zhu et al. (2022)
33	*Rhabdophis leonardi* (Clade C in Zhu et al. (2022))	China	HT0851	LC325747	LC325300	Takeuchi et al. (2018)
34	*Rhabdophis murudensis*	Malaysia	HT0788	LC325787	LC325341	Takeuchi et al. (2018)
35	*Rhabdophis nigrocinctus*	Shan State, Myanmar	CAS215280	KF800926	KF800936	Zhu et al. (2014)
36	*Rhabdophis nuchalis*	Shennongjia, Hubei, China	SICAU090001	KF800925	KF800935	Zhu et al. (2014)
37	*Rhabdophis pentasupralabialis*	Jiulong, Sichuan, China	GP1065	KF800924	KF800934	Zhu et al. (2014)
38	*Rhabdophis plumbicolor*	Sri Lanka	SL‐Mp‐1	OK315953	OK315841	Deepak et al. (2022)
39	*Rhabdophis plumbicolor*	Sri Lanka	HT0783	LC325783	LC325337	Takeuchi et al. (2018)
40	*Rhabdophis plumbicolor2*	Sri Lanka	HT0784	LC325784	LC325338	Takeuchi et al. (2018)
41	*Rhabdophis helleri*	Baoshan, Yunnan, China	RDQ200905366	KF765393	KF765398	Zhu et al. (2014)
42	*Rhabdophis helleri2*	Panzhihua, Sichuan, China	SCUM090014	KF765394	KF800927	Zhu et al. (2014)
43	*Rhabdophis helleri3*	Hong Kong, China	SICAU090029	KF800918	KF800928	Zhu et al. (2014)
44	*Rhabdophis swinhonis*	Taiwan, China	KUZR 18977	AB861888	AB842176	Zhu et al. (2014)
45	*Rhabdophis tigrinus*	Japan	HT0098	LC325751	LC325305	Takeuchi et al. (2018)
46	*Rhabdophis tigrinus2*	Japan	HT0177	LC325752	LC325306	Takeuchi et al. (2018)
47	*Xenochrophis maculatus*	Malaysia	HT0720	LC325335	LC325781	Takeuchi et al. (2018)
48	*Xenochrophis trianguligerus*	Malaysia	HT0795	LC325344	LC325790	Takeuchi et al. (2018)
49	*Xenochrophis vittatus1*	Indonesia	HT0615	LC325768	LC325322	Takeuchi et al. (2018)
50	*Xenochrophis vittatus2*	Unknown locality	FMNH 257460	EF395920	EF395895	Alfaro et al. (2008)

Our phylogeny was compared especially to several recent phylogenies of Asian natricines. We have relied heavily on the more expansive analysis of the *Rhabdophis nuchalis* Group by Zhu et al. (2022), which included DNA from several of the specimens of *Rhabdophis leonardi* described here, as did Piao et al. (2020). Deepak et al. (2022) included a sample identified as *R. leonardi*, whereas Das et al. (2021) did not.

### 2.2 Morphological data

The holotype of *Natrix leonardi* Wall, 1923, once located, was closely examined at its repository, and the data were compared to the original description to verify its identity. Photographs of the holotype were taken by Kevin Webb of the Photo Unit of the Natural History Museum, London.

The color pattern of several Chinese specimens in life and following preservation were obtained from field notes, photographs, and direct observations. Sex was determined by the presence or absence of hemipenes and by inspection of gonads. The right hemipenis was everted in the only male specimen (SICAU201705031) available to us, using the method described by Jiang (2010), and described using the terminology of Dowling and Savage (1960). Skulls of two specimens (the male and one female) were scanned using a 225 kV microcomputerized X‐ray tomography (microCT) unit at the Key Laboratory of Vertebrate Evolution and Human Origins of the Chinese Academy of Sciences (CAS).

Measurement methods and definitions follow Zhu et al. (2014). The following features were measured with a ruler to the nearest 1 mm (abbreviations in parentheses): total length (TL); snout‐vent length (SVL); tail length (TaL). Measurements made with calipers, to the nearest 0.1 mm, included: head length (HL: from the snout tip to the posterior margin of the mandible); head width (HW: measured at the widest part of the head), and eye width (EW: maximum horizontal eye width); The following scale characters and tooth counts were also recorded, following Zhao (2006): supraoculars (SPO); preoculars (PRO); postoculars (PTO); loreals (LR); temporals (TEM); supralabials (SL); infralabials (IL); ventrals (VEN); dorsal scale rows (DSR); subcaudals (SC); maxillary teeth (MT).

Morphological data for most congeners were obtained from the literature (Boulenger, 1893, 1900; Bourret, 1935; Das, 2010; Das et al., 2021; David & Vogel, 2010, 2021; de Lang & Vogel, 2006; Günther, 1858, 1864; Jiang & Zhao, 1983; Leviton, 1970; Ota & Mori, 1985; Smith, 1943; Stuebing & Lian, 2002; Takeuchi, 2021; Tweedie, 1957; Zhao, 1997; Zhao & Adler, 1993; Zhao et al., 1998; Zhu et al., 2014). Specimens of *R. leonardi* that were examined are listed in Appendix 1.

### 2.3 Incubation

Four eggs were laid by one of the females described herein (SICAU201705027) and were incubated at Sichuan Agricultural University (Chengdu, Sichuan Province, China), at 25°C. Only one hatchling emerged.

### 2.4 Collection catalog prefix abbreviations

BMNH: Natural History Museum, London (Now also NHMUK: National History Museum of the United Kingdom, London); BNHS (formerly BNHM): Bombay Natural History Society/Museum, Mumbai; KIZ: Kunming Institute of Zoology, Chinese Academy of Sciences, Kunming, China; RDQ: Ding‐qi Rao, Field tag, Kunming, China; SICAU: Sichuan Agricultural University, Ya'an, China; SCUM: Sichuan University Museum, Chengdu, China.

## 3 RESULTS

### 3.1 Molecular phylogenetic relationships of *Rhabdophis leonardi*

**FIGURE 1:**
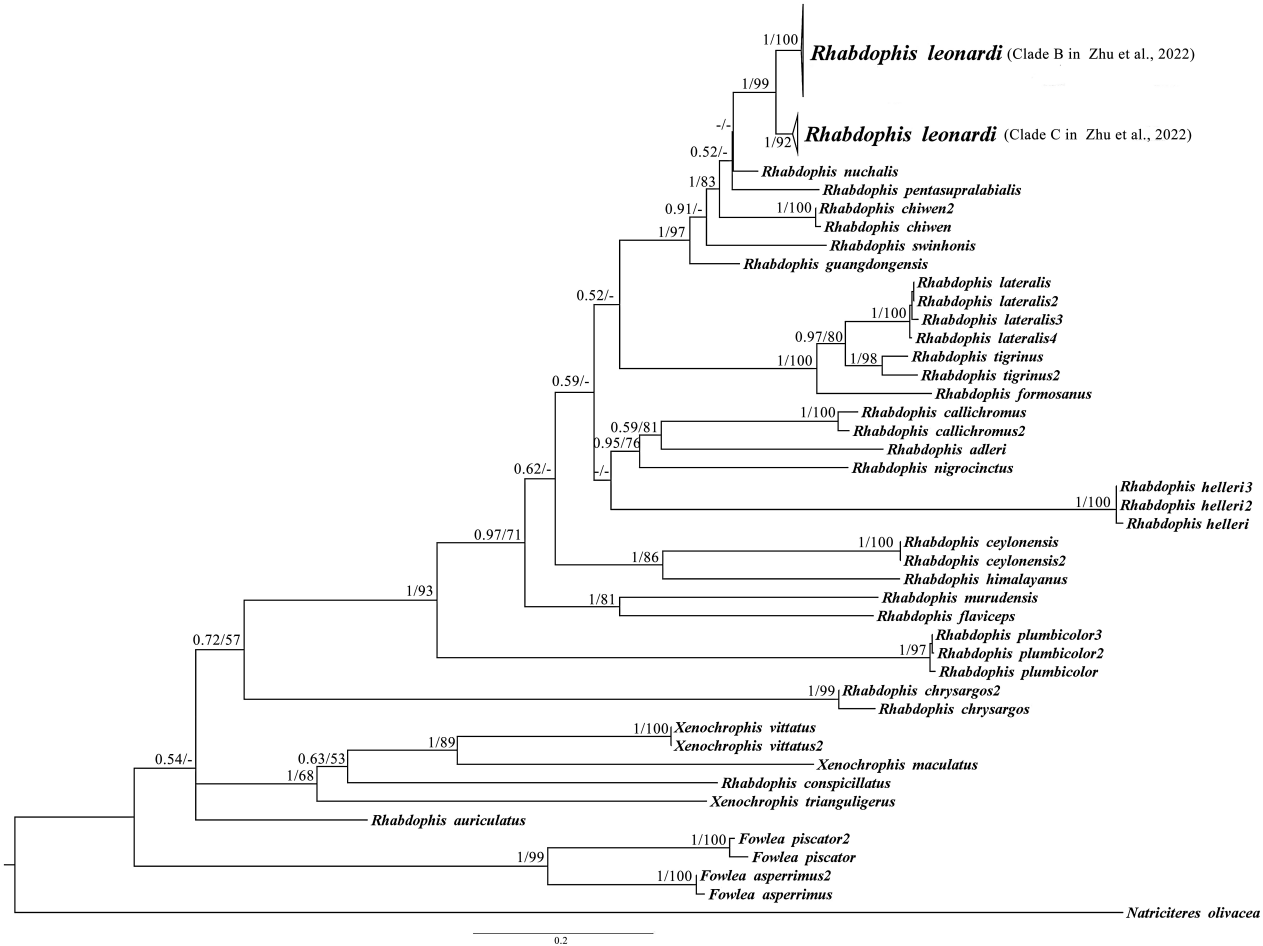
Bayesian inference (BI) phylogenetic tree based on concatenated cyt *b* and *c‐mos* gene sequences. The phylogenetic tree includes the Bayesian posterior probability (PP) and maximum likelihood bootstrap (BS) values of the nodes (PP/BS). A hyphen (−) indicates BS < 50. The scale represents sequence dissimilarity, or the percentage of base substitutions; the length of the scale in the figure represents 20% dissimilarity in sequences.

The topology of the maximum likelihood (ML) tree was consistent with that of the Bayesian inference (BI) tree (Figure 1). With respect to the position of *Rhabdophis leonardi* and its substructure, our phylogenetic results largely conform to those of Zhu et al. (2022), who focused on relationships among the *R. nuchalis* Group, to which *R. leonardi* belongs.

### 3.2 Type locality of *Natrix leonardi*

In 1923 Frank Wall reported on a collection of snakes from Sinlum Kaba, in the Upper Burma Hills. The collection included a single specimen of a new species of natricine snake, described by Wall as *Natrix leonardi*. This was not the first collection made by Mr. Leonard in that locality. An earlier collection of snakes from the same locality had been published upon by Wall in 1921, including a new ratsnake described as *Coluber leonardi*. The description of two snakes from the same locality by the same author within 2 years of each other, and bearing the same patronym as the specific epithet, understandably led to confusion regarding the type specimens. The brevity of the description of *N. leonardi*, and in particular the absence of information on the sex of the holotype, resulted in further confusion regarding the application of the name. Finally, the type locality itself presents several complications. Wall provided different coordinates for the site in his 1921 and 1923 reports. Sinlum Kaba itself has several spellings and appears in two physical locations on older maps, whereas it does not appear at all in searches of recent online maps.

In the 1920s P. M. R. Leonard, a member of the Burma Frontier Service, traveled repeatedly to the region near the Chinese border. By the time a survey of the aquatic mollusks had been conducted in the region, to assess the presence *Schistosoma*, Leonard was cited in the acknowledgments as Assistant Superintendent of the Northern Shan States, serving at Kutkai (Rao, 1928). Today, Kutkai is approximately 240 km by road (via Highway 3) southeast of Bhamo, the closest major city to the type locality (Google Maps, 2022) and, even in colonial times, an important port along with the Irrawaddy (or Ayeyarwady) River.

Wall (1923) described the origin of the specimens, including the holotype of *Natrix leonardi*, as “Sinlum Kaba, Upper Burma Hills, circ. 6,000 feet (Lat. 25^o^, Long. 97^o^).” Importantly, he does so without reference to his 1921 paper, in which he gives the coordinates for Sinlum Kaba as “Lat. 24^o^ Long. 97^o^.” Indeed, the coordinates in Wall's, 1921 paper are the closer of the two to the true type locality. Even rounding to the nearest degree, the coordinates in Wall's, 1923 description of *N. leonardi* not only place the locality farther north, but also on the western side of the Irrawaddy River, in or near the river's floodplain. The 1921 coordinates place the locality southwest of Bhamo and south of a bend in the Irrawaddy River, again in the floodplain. In contrast to the publications, the catalog data for the type specimen (see below) place Sinlum Kaba quite accurately as 24.27° N, 97.52° E. (Wallach et al., 2014, also noted the inconsistencies in Wall's descriptions of this locality, but they did not attempt to resolve them.)

The type locality, Sinlum Kaba, lies in a triangular mountainous region above and to the east of Bhamo, in southeastern Kachin State. Recently recorded family history, published in a series of articles in the *Katchinland News*, sheds some light on the still confusing history of Sinlum Kaba (Lahpai, 2017, 2020; Pangmu, 2011), from which the following account is synthesized. From those published clan histories, it appears that Sinlum Kaba was established by the Gauri Lahpai tribal leader Zau Bawm in the early to mid‐1800s (Lahpai, 2017). By the late 1800s Baptist missionaries William Henry Roberts and Ola Hansen had established a church and school in Bhamo, where they baptized Zau Tu, a member of the Gauri Lahpai chieftain clan, and Hka Jan (Lahpai, 2017; Pangmu, 2011). That couple married and proceeded to establish a mission in their ancestral homeland, in the mountains near the Chinese border. The couple cleared an area in the forest and established a new settlement, which they named Pangmu, located only about 24 km straight‐line distance from the Chinese border, which lay between Lwegel, Myanmar, and Laying, Longchuan County, Yunnan Province, China.

Shortly thereafter, seeing the strategic value of the location with respect to the international border, the British colonial authorities commandeered the settlement to use as an administrative center and ordered Zau Tu and Hka Jan to relocate (Pangmu, 2011). The original settlement, now under British authority, became known as Sinlum Kaba, while Zau Tu and Hka Jan established a new settlement about 1.6 km away, which they again named Pangmu (Pangmu, 2011). The British outpost grew in importance, with a fortified military station, a center for handling legal matters involving the Kachin people, and plantings of fruit trees imported from England (Lahpai, 2020). Meanwhile, a high school that had been established in Pangmu by Zau Tu and Hka Jan in 1903 was moved to Sinlum Kaba in 1928. Lahpai (2020) notes that Hka Jan was recognized for her contributions as an educator at a Durbar (a gathering of regional dignitaries) in Sinlum Kaba in 1923. Such an event may well have involved a visit by colonial authorities such as Leonard, affording him an opportunity to acquire the specimens reported that year by Wall.

A search for “Sinlum Kaba” or “Sinlumkaba” in current online maps (Google Maps or Google Earth) fails to return any records. However, a 1:250,000 topographic map (https://www.oldmapsonline.org/map/harvard/009402828‐47) (sheet “Lung‐Ling, China; Burma” https://iiif.lib.harvard.edu/manifests/view/ids:2402727) compiled by the U.S. Army Map Service in 1954 shows two villages labeled “Sinlumkaba” less than 3 km apart, with “Pangmo” about halfway between them. Currently the two largest clearings in that area, judging from the satellite image on Google Maps (2022 imagery), are located at approximately 24.243323 N, 97.498559E and at 24.265404 N, 97.498880E. Clicking on either of those sites returns the label “Sing Lun.” Furthermore, searching on “Sing Lun” returns a political boundary (presumably municipal) that encompasses both clearings, which are located just over 2 km apart. Both clearings appear to contain substantial settlements, including several large structures, especially at the southern site, which also appears to include a helipad. These two clearings presumably represent the two “Sinlumkaba” sites on the topographic map. Approximately 1 km to the NE, at 24.269804^o^N, 97.510097° E, a click returns the label “Pan Mu,” and a similar municipality labeled “Pan Mu” appears to the northeast, sharing a border with Sing Lun. Descendants of Zau Tu and Hka Jan reportedly gathered at the Sinlum Church as recently as 2011, and the couple's home in Pangmu “is now considered a historic and heritage site” (Pangmu, 2011).

**FIGURE 2:**
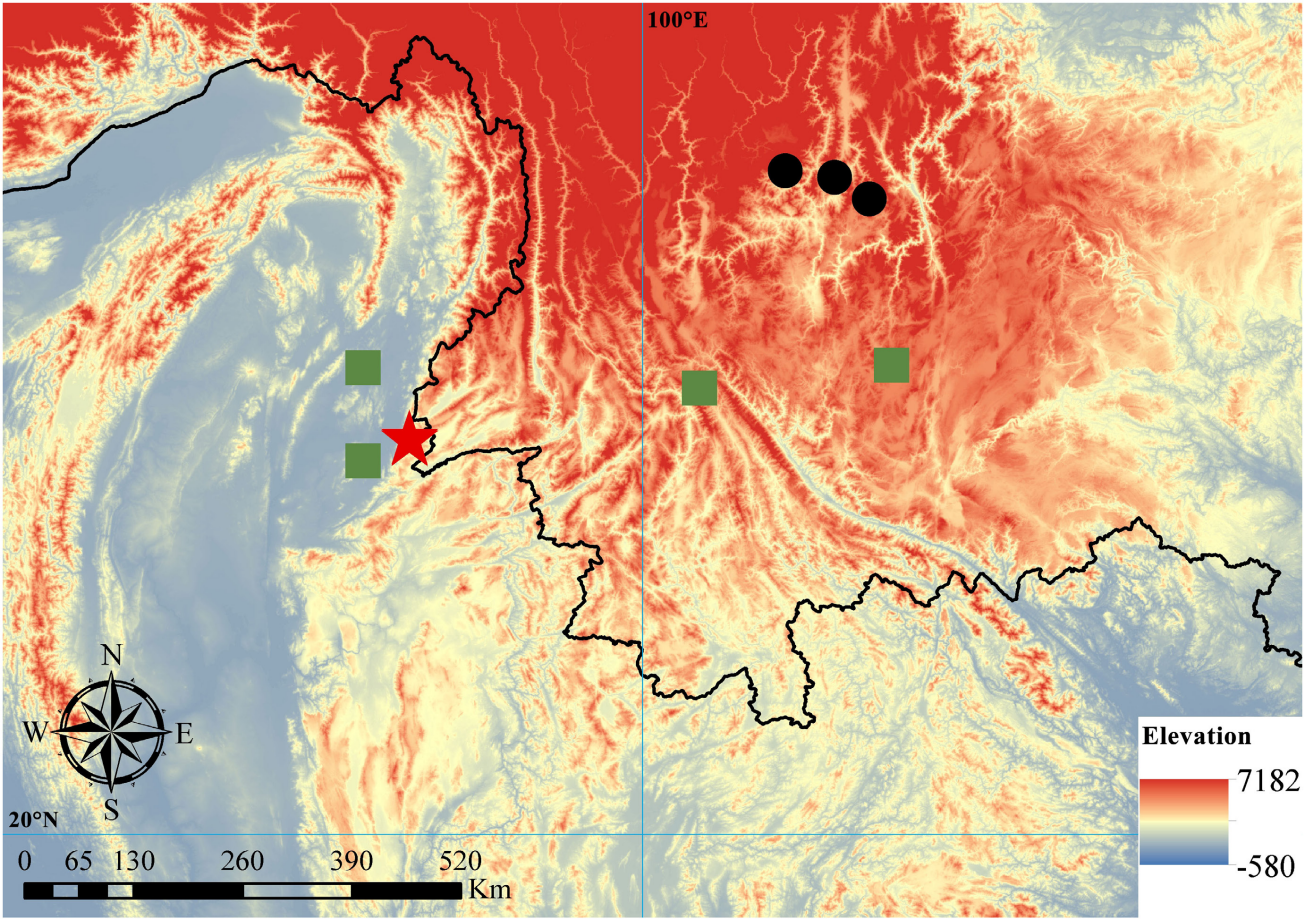
Map showing the localities of *Rhabdophis leonardi* in southern Sichuan and Yunnan Provinces, China, and the type locality in Myanmar. Red star represents the type locality (Sinlum Kaba, Myanmar). Black circles represent the localities for specimens examined in this study, including Xiaoshanbao Village and Huangcao Village from Liangshan Yi Autonomous Prefecture, and Hongbao Village from Panzhihua City. Green squares represent the locality of referred material, including two localities described in Wall (1921, 1923), Wuliang Mountain, and the mountains west of Kunming. (Map generated with ArcMap 10.2 (ESRI, Inc.) with WGS84 datum and Universal Transverse Mercator conformal projection).

In summary, the type locality of *Natrix leonardi*, Sinlum Kaba, can confidently be placed in the mountains east of Bhamo, Kachin State, at approximately 24.24–24.26^o^N, 97.50E (Figure 2). It is not clear from either of Wall's papers whether the specimens were in fact procured close to Sinlum Kaba itself or from the wider surrounding area.

### 3.3 Location of the holotype

**FIGURE 3:**
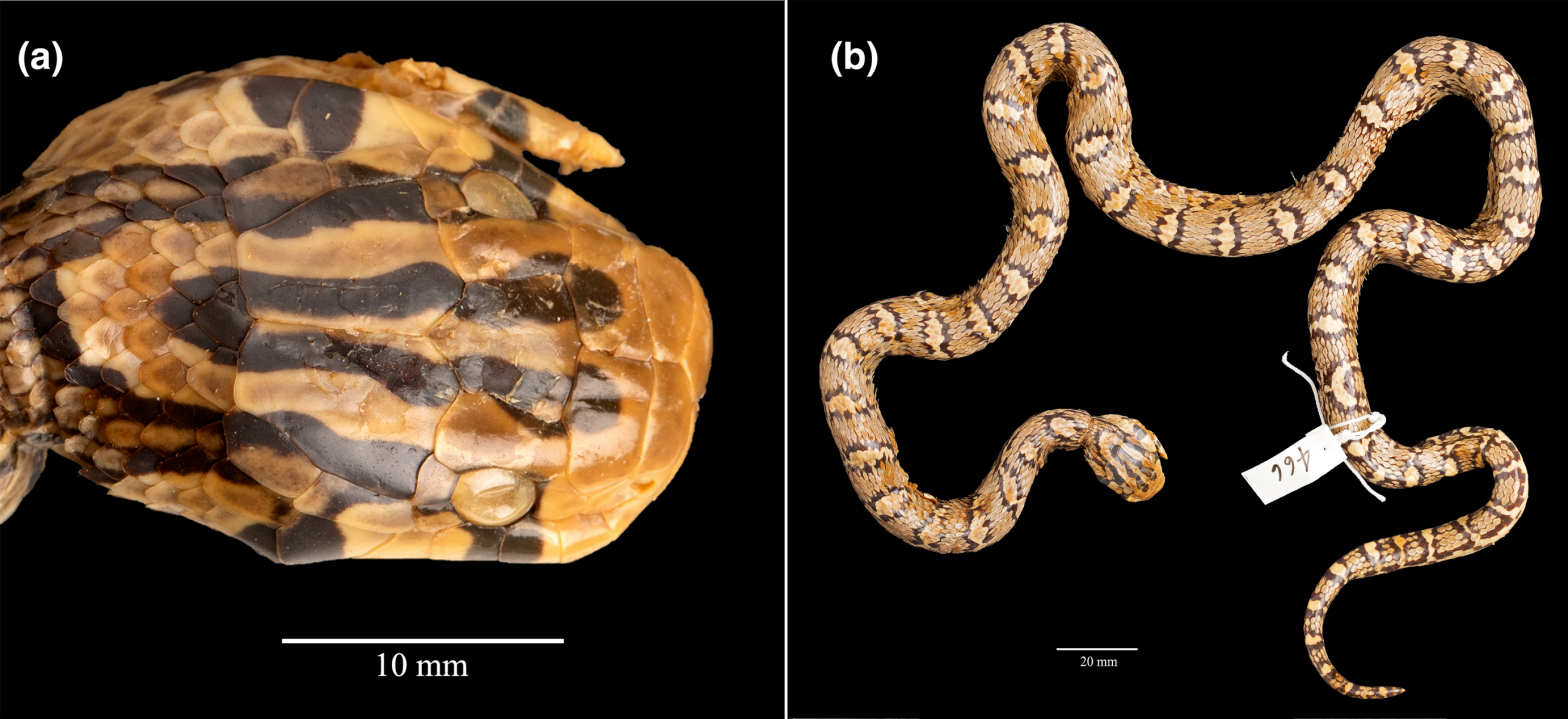
Photos of BNHS 466 (formerly BNHM 466, photos by Rahul Khot), a specimen of *Archelaphe bella*. (a) dorsal view of head; (b) dorsal view of specimen. This specimen has been variously cited in the literature as the holotype of *Coluber leonardi* (Smith 1921) (now *Archelaphe bella*) and of *Natrix leonardi* (Smith 1923) (now *Rhabdophis leonardi*). The specimen is definitively not the holotype of *Natrix leonardi* and may not be the holotype of *Coluber leonardi*. See text for a full discussion of this specimen.

Recent information on the location and identity of the holotype of *Natrix leonardi* presented similar ambiguities. Both of Wall's reports on collections of snakes from Sinlum Kaba (Wall, 1921, 1923), each describing one new species of snake as a patronym for P. M. R. Leonard, imply that the specimens were donated to the Bombay Natural History Society (BNHS), whose museum collection was earlier recognized by the acronym BNHM (Leviton et al., 1985). Since 2020 the standard acronym of the BNHS museum collection is BNHS, and the acronym BNHM is now obsolete (Sabaj, 2020). However, only one of the holotypes of those two species has been specifically described in the literature as being deposited at the BNHS (Das & Chaturvedi, 1998), and even that designation may be in error. Importantly, the Reptile Database cites the holotype of *Natrix leonardi* as “BNHS = BNHM 466” (Uetz et al., 2022; accessed 25 March 2023). One of us (RK) examined and photographed that specimen and found it to be a colubrine snake, not a natricine. Indeed, that specimen is listed in the BNHM (now BNHS) type catalog (Das & Chaturvedi, 1998) as the holotype of *Coluber leonardi* (Wall, 1921; Figure 3), described in the *Journal of the Bombay Natural History Society*, and now considered a synonym of *Archelaphe bella* (Schulz et al., 2011). We note, however, that BNHM 466 appears in the photograph to be considerably larger than the holotype of *Coluber leonardi* (279 mm SVL; Wall, 1921). We suggest that BNHM 466 may, in fact, be the third and much larger specimen of *Coluber leonardi* (685 mm SVL), which was reported by Wall (1923) in the same paper in which he described *Natrix leonardi*. Thus, the identity and repository of the holotype of *Coluber leonardi* remains to be clarified, but resolution of that question is beyond the scope of this report.

**FIGURE 4:**
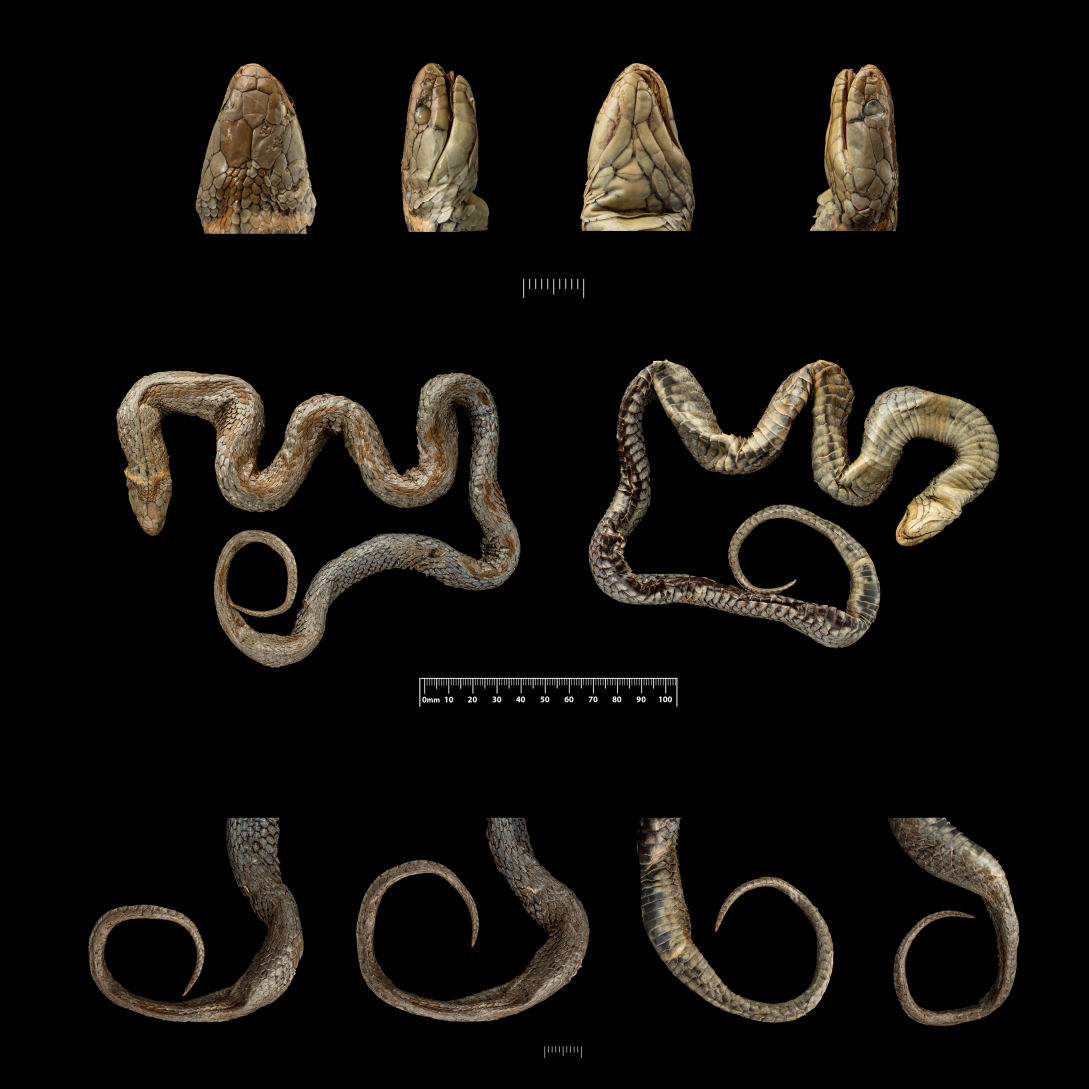
Photos of the holotype of *Rhabdophis leonardi*, BMNH 1946.1.12.86 (female). Top row (left to right), details of head in dorsal, right lateral, ventral, and left lateral views. Center row (left to right), dorsal and ventral views of entire specimen. Bottom row (left to right), details of posterior body and tail in dorsal (2 views), ventral, and left lateral views. (photos by Kevin Webb, Photo Unit, Natural History Museum, London).

Because the holotype of *Natrix leonardi* was not found in the BNHM and was not listed in the type catalog for that collection, we next examined the holdings of the Natural History Museum, London (formerly British Museum (Natural History)), whose catalog number prefix for historical material is BMNH, and identified a specimen of *Rhabdophis leonardi*, BMNH 1946.1.12.86 (formerly BMNH 1923.10.13.39), from the Upper Burma Hills (Figure 4). Upon examination by two of us (DG and VD) and comparison with Wall's (1923) description, that specimen was determined to be the holotype of *N. leonardi* and, indeed, it was listed as a “type” in the BMNH catalog. As with other type specimens in the London collection, that specimen was re‐cataloged and assigned a new number after the end of World War II when specimens were returned from safekeeping.

Although designated as a type specimen and bearing the more accurate coordinates for the type locality than those published by Wall (1921, 1923), BMNH 1946.1.12.86 does not appear to have been identified previously in print as the holotype of *Natrix leonardi*. Parker (1925) referenced two specimens from “Sinlum Kaba, Upper Burma,” one of them described as the “Type” of *Natrix leonardi* and the other as doubtfully attributed to that species. Both presumably were under his curation at the BMNH, although no catalog numbers were provided.

If indeed BNHS 466, in Mumbai, is the holotype of *Coluber leonardi*, then why that specimen and the holotype of *Natrix leonardi*, both procured from Sinlum Kaba 2 years apart, found their way to different repositories over 7000 km apart is not clear. Alternatively, the holotype of *Coluber leonardi* may similarly have made its way to the British Museum or another institution, and the specimen in Mumbai may be incorrectly identified as the type. Perhaps adding to the confusion over the identity and repositories of the two type specimens is the fact that the recommended acronym for the herpetological collection of the Bombay Natural History Society, BNHM, is an anagram of that for the Natural History Museum, London, BMNH (Leviton et al., 1985). Furthermore, the catalog number of the presumed holotype of *Coluber leonardi* in Mumbai is BNHM 466 (Das & Chaturvedi, 1998), which coincidentally is the same number as the page on which the description of *Natrix leonardi* appeared 2 years later, in the same journal (Wall, 1923).

### 3.4 Partial redescription of the holotype of *Natrix leonardi*

The original description of *Natrix leonardi* (Smith, 1923), based on a single specimen, provides sufficient detail to confirm with confidence that the holotype is BMNH 1946.1.12.86 (formerly BMNH 1923.10.13.39), shown in Figure 4. The previous number indicates that the specimen was first cataloged on 13 October 1923, the year of Wall's description of the species, and the current number indicates that the specimen was re‐cataloged on January 12, 1946, after specimens were returned to the museum from safekeeping off‐site following World War II. That specimen was examined by two of us (DG and VD), with specific attention to features missing or inconsistent with the original description, which is lacking in some important details.

Importantly in this sexually dimorphic species, the holotype is a female, with a snout‐vent length of 604 mm and a tail length of 122 mm. Neither the sex nor the size of the holotype was reported by Wall (1923). The habitus of the holotype is robust, with a relatively wide head. A narrow pale collar is present, two scale rows wide (6–7 rows behind the parietal scales). The pale orange collar presumably has faded in preservative.

Several scale counts differ from the original description, including the number of ventral scales, which Wall (1923) reported as 152. Two independent counts (by DG and VD) obtained a count of 150 using the Dowling (1951) method, which, while less subjective than earlier methods of counting (such as simply counting from the first markedly elongated scale), often returns a slightly lower number. The supralabial scales are 6 on both sides, rather than the 7 reported in the original description. Wall himself noted this, stating “*Supralabials*. 7. (The 5th and 6th are confluent making them appear 6).” Our observations indicate that Wall's “5th and 6th” are indeed a single scale (clearly evident in Figure 4), so the correct supralabial count is 6. The infralabials are 8 on both sides, not 6 as reported by Wall.

The most important feature of the scutellation is the number of midbody scale rows, which may variously be counted as 15 or 17. The reason is that the dorsal scale rows reduce, in the holotype, through loss of row 4, at the level of the 74th ventral on the left side and 76th on the right side. That lies at approximately 49–51% of the total ventral count. Including the additional length of the head, it is likely that the higher dorsal scale row count of 17 would be made at midbody, although an individual casually selecting a point at the midbody might reasonably count either 15 or 17 dorsal scale rows.

We note that Parker (1925), in a study of dorsal scale row reduction in specimens he identified as *Natrix nuchalis*, placed the reduction from 17 to 15 dorsal rows at approximately 45% of “Body Length” in the “Type” of *Natrix leonardi* (presumably then cataloged as BMNH 1923.10.13.39) and at over 50% of “Body Length” in a second specimen recognized, doubtfully, as the same species. Importantly, Parker did not define “body length,” but he distinguished the body from a region he identified as the “neck” (but also did not define). Parker considered that all the species he examined were conspecific with what is now *Rhabdophis nuchalis*, but that conclusion has not been supported by recent analysis of that complex species group (Zhu et al., 2022).

### 3.5 Diagnosis and description of recent material from Sichuan Province, China

#### 3.5.1 Diagnosis

*Rhabdophis leonardi* is characterized by the following combination of features: (1) adults with a distinct annulus (collar) around neck, broad and red in males, narrow and orange with irregular black borders in females; (2) dorsal ground color pale green or olive; (3) nuchal groove present, bordered by enlarged scales; (4) some lateral and dorsal scales with black edges, their frequency gradually increasing from anterior to posterior, eventually forming irregular and indistinct transverse bands; (5) body size relatively large, adults approximately 600–700 mm; (6) tail moderate, longer in males (the ratio of tailL/TL is approximately 14.7% to 15.6% in females; approximately 19.7% in male); (7) nostril large and lateral, nasal scale completely divided; (8) eye with notably green iris; (9) SL 6, the 3rd and 4th in contact with the eye, with a conspicuous black oblique band across the suture between the 4th and 5th supralabials; (10) IL usually 7, the first pair in contact behind the mental scale, first 4 in contact with anterior chinshields, and 4th to 6th in contact with posterior chinshields; (11) PRO usually 1; (12) PTO 3; (13) TEM usually 1 + 2; (14) DSR 17–17/15–15 (with reduction from 17 to 15 rows occurring near midbody, at approximately ventrals 74–77), strongly keeled except the second row (weakly keeled) and the first row (smooth); (15) VEN 145–155; (16) anal divided; (17) SC approximately 40–60, paired; (18) MT 19 (16 + 3), posterior maxillary teeth only slightly enlarged and ungrooved.

#### 3.5.2 Description of adult male

**FIGURE 5:**
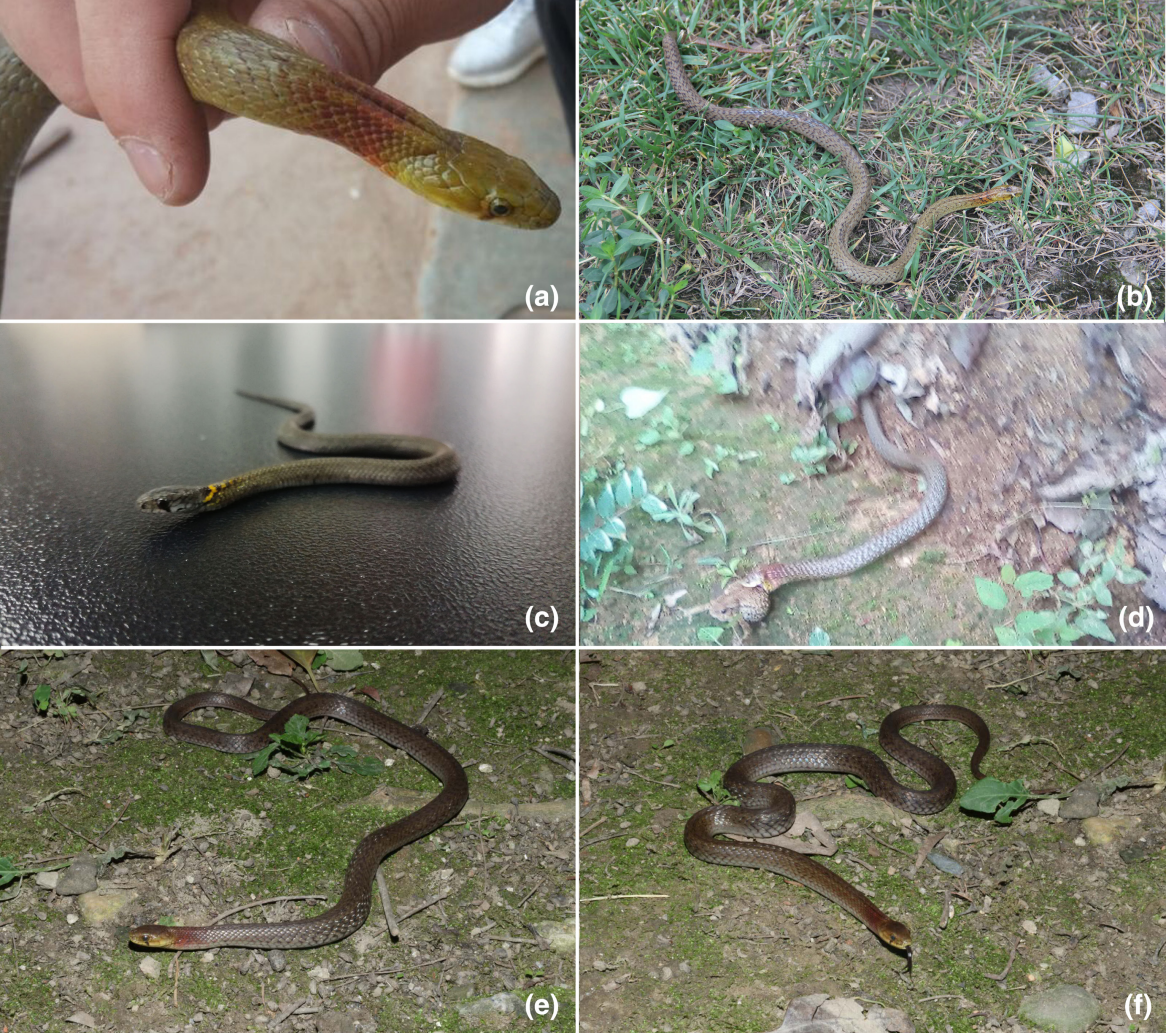
Photos of *Rhabdophis leonardi* in life. (a) Male (SICAU201705031) (photo by Shijun Yang); (b) Female (SICAU201705027) (photo by Guangxiang Zhu); (c) offspring of SICAU201705027, incubated in laboratory (SICAU201707021) (photo by Wenjiang Tang); (d) out‐of‐focus photo of *R. leonardi* consuming a toad in the field (photo by Mr. Xu from Huangcao Village, Panzhihua City, China); (e, f) individual (not collected) from the West Mountain of Kunming, Yunnan Province, China (photos by Li Ding).

**FIGURE 6:**
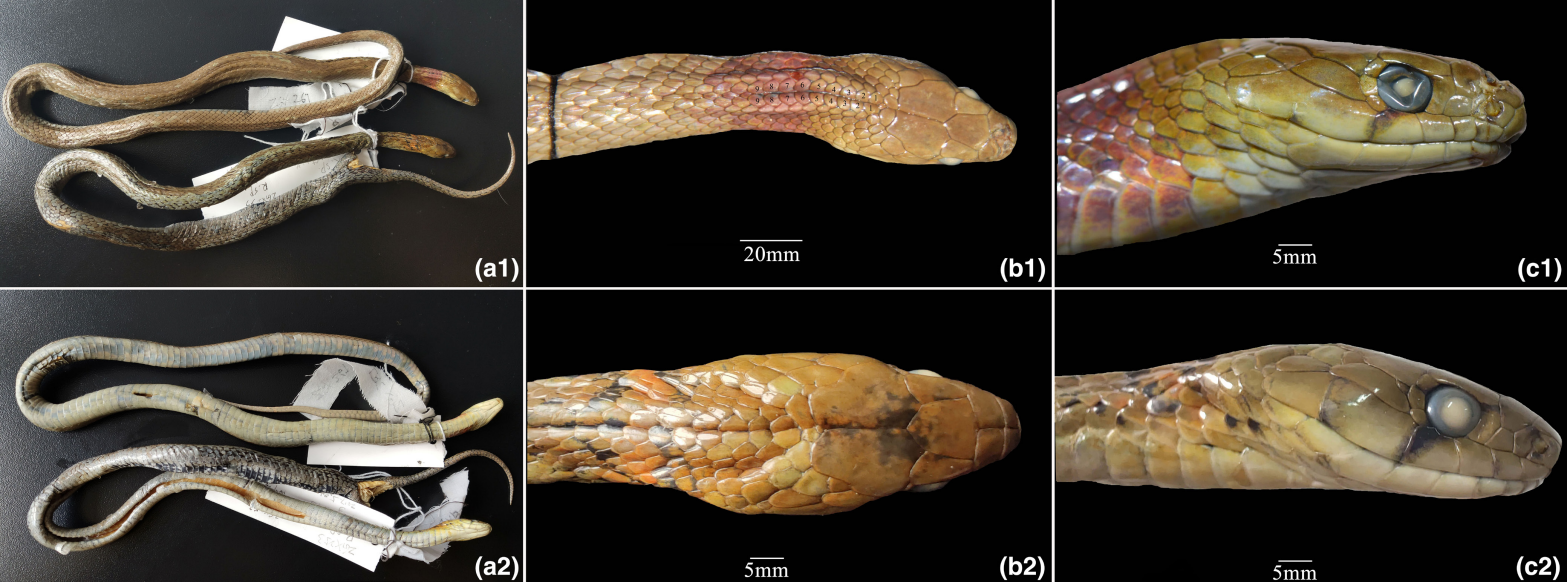
Photos of *Rhabdophis leonardi* in preservative. (a1) dorsal view of male (SICAU201705031), top, and female (SICAU201705027), bottom; (a2) ventral view of male (SICAU201705031), top, and female (SICAU201705027), bottom; (b1) dorsal view of head of male (SICAU201705031; note broad red collar and the nuchal groove); (b2) dorsal view of head of female (SICAU201705027; note narrow orange collar with irregular black borders and the nuchal groove); (c1) lateral view of head of male (SICAU201705031); (c2) lateral view of head of female (SICAU20170502). Photos by Guangxiang Zhu and Shijun Yang.

SICAU201705031, collected from Xiaoshanbao Village (26.808889° N, 102.434167° E; 2230 m elevation, datum = WGS84), Huili County, Liangshan Yi Autonomous Prefecture, South Sichuan Province, China, on May 31, 2017 (Figures 5, 6).

*Size*: TL 674 mm (SVL 541 mm and TaL 133 mm), TaL/TL 19.7%.

*Head*: Moderately large and distinctly wider than neck, HL 17.55 mm, HL/TL 2.6%; HW 12.04 mm. Eye large, EW 3.81 mm, EW/HL 21.7%. Rostral semicircular and much broader than deep, just visible from above. Nostril large and lateral, in a completely divided nasal scale. Internasals slightly shorter than prefrontals. Prefrontals wide, no distinct canthus rostralis. Frontal slightly longer than broad, as long as the distance from the rostral to frontal. LR single, longer than deep. SPO single; PRO single; PTO 3; subocular absent. SL 5/6, the 5th incompletely divided on the left, the 1st divided, and the last two scales on the right, so the 3–4th/4–5th in contact with the eye; a conspicuous oblique black band on the suture between the 4th/5th supralabial on each side. IL 7/8, the first pair in contact with each other behind the mental scale, the first four in contact with anterior chinshields, the fourth to sixth in contact with posterior chinshields, TEM 3 (1 + 2).

*Trunk*: Round in cross‐section. Nuchal groove present, with nine distinctly enlarged scales on either side. Dorsal scale rows 17–17‐15, all strongly keeled, except the second row feebly keeled and the first row smooth. Reduction from 17 to 15 rows occurs at level of ventrals 77/75. Ventrals 149.

*Tail*: Moderate in length. Anal divided. SC 58, paired. Scale row reduction formula on the tail:

$$12\frac{4\left(2+3\right)}{4\left(2+3\right)}10\frac{7\left(2+3\right)}{1\left(1+2\right)}8\frac{23\left(3+4\right)}{24\left(3+4\right)}6\frac{43\left(2+3\right)}{41\left(2+3\right)}$$

*Coloration*: Dorsal ground color of SICAU201705031, a male, in life was pale green, with a wide red collar on the neck that extended for approximately six scales (Figure 5a). The venter had black spots with red edges from the anterior body to halfway along with tail, the density increasing to midbody and then decreasing. The colors gradually faded in preservative, with the red nuchal band turning to pale red (Figure 6).

**FIGURE 7:**
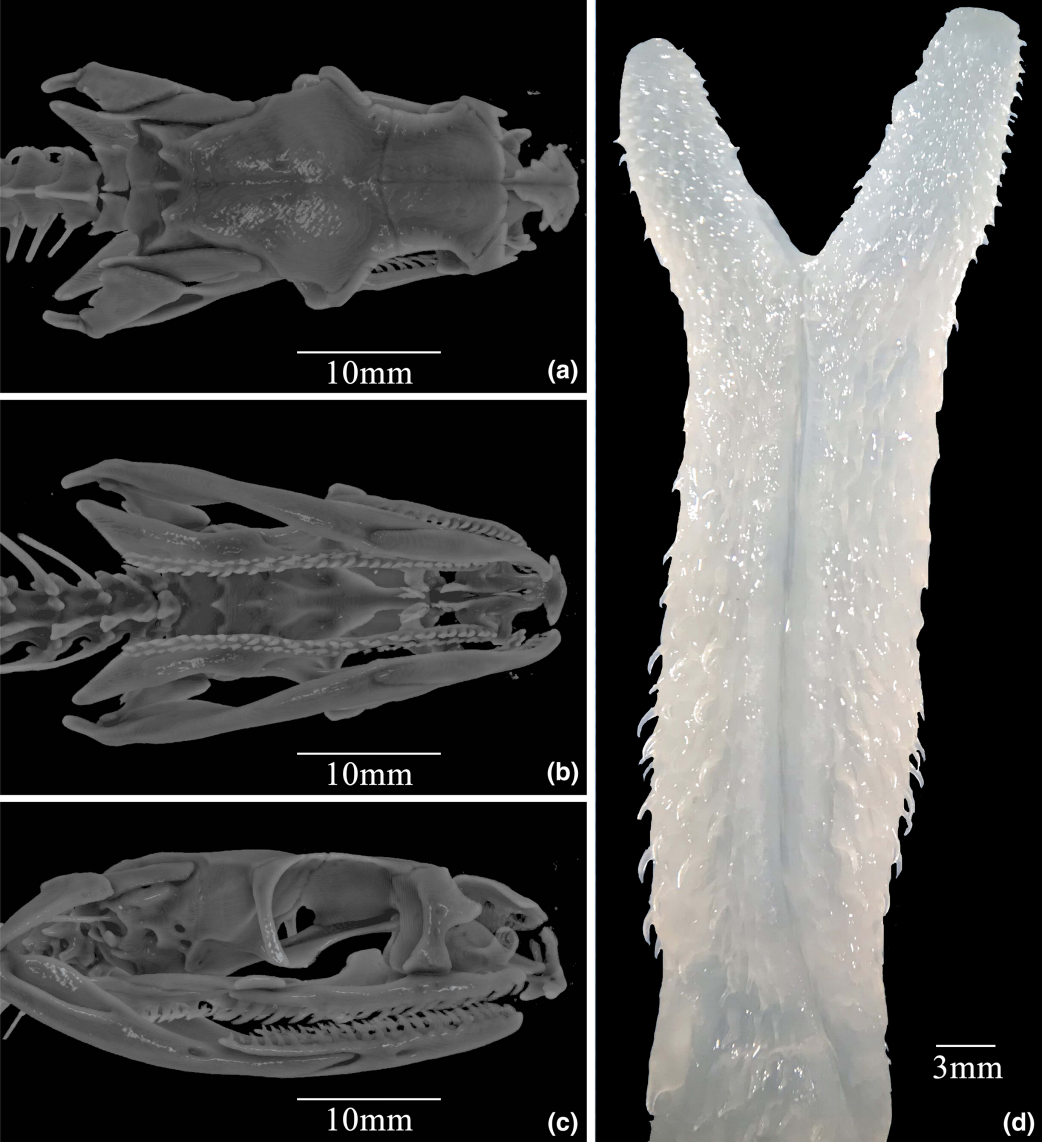
MicroCT scan of skull (a–c) and photo of hemipenis (d) of male *Rhabdophis leonardi* (SICAU201705031). (a) dorsal view; (b) ventral view; (c) lateral view; (d) sulcate side.

*Skull*: The skull of SICAU201705031 is illustrated in Figure 7. It generally resembles the skull of other natricine snakes, although several features (e.g., a relatively robust quadrate bone) resemble those of species with less fully surficial habits. Nineteen maxillary teeth are present on both sides; the last two are slightly enlarged (and to a much lesser degree, the third from last), and are ungrooved (Figure 7).

*Hemipenis*: The retracted hemipenis extends to the ninth subcaudal, bifurcating at the level of the sixth subcaudal. The everted hemipenis is capitate and bilobed. Most of the organ is covered with small, hooklike spines, larger toward the base, with one large basal spine opposite the sulcus spermaticus. The centripetal sulcus extends halfway to the tip of each lobe. The prominent edges of the sulcus are smooth (Figure 7).

#### 3.5.3 Description of females

Morphological data for the other female specimens are listed in Table 2 and Figures 5, 6. The female specimens differ most importantly from the male in the size and color of the nuchal collar. In adult females the collar is narrower (extending for about three to four scales) and is orange with narrow, irregular anterior and posterior black borders (Figure 6). Although our sample is small, we believe this represents sexual dimorphism in the color of the nuchal collar, a feature that should be confirmed by future studies. The female hatchling generally resembles the adult females in nuchal coloration, although the collar is yellow rather than orange, contrasting more strongly with its prominent black borders (Figure 5c).

**TABLE 2:** Measurements and scale counts for five individuals of *Rhabdophis leonardi.* See Materials and Methods for abbreviations of characters.

Sample no.	Location	TL (mm)	TaL (mm)	SPO	PRO	PTO	LR	TEM	SL	IL	VEN	DSR	SC	Sex and note
SICAU201705031	Xiaoshanbao Village	674	133	1	1	3	1	1 + 2	5/6	7/8	149	17–17‐15	58	Male
SICAU201705027	Hongbao Village	598	99	1	1	3	1	1 + 2	6	8	150	17–17‐15	49	Female
SICAU201807001	Huangcao Village	615	96	1	1	3	1	1 + 2	6	8/7	152	17–17‐15	44	Female
SICAU201705026^a^	Xiaoshanbao Village	Unmeasured	Unmeasured	1	1	3	1	1 + 2/1 + 1 + 2	6	8/7	Unmeasured	Unmeasured	41	Female
SICAU201707021	‐	166	38	1	1	3	1	1 + 2	5	8	145	Unmeasured	63	Female, hatchling^b^

a Found dead on road; not all features intact.

b hatchling from egg laid by SICAU201705027 on June 4, 2017 at Sichuan Agricultural University, Chengdu, Sichuan Province, China.

#### 3.5.4 Comparisons

The number of dorsal scale rows in *R. leonardi* (17–17/15–15) distinguishes it from all congeners except *R. auriculatus* (Table 3), in which the scale rows reportedly also are reduced from 17 to 15 at about the midbody (Leviton, 1970). However, *R. auriculatus*, which is endemic to the Philippines, has white lines on the sides of the body and behind the eyes, as well as white spots on the dorsum.

**TABLE 3:** Significant characters of *Rhabdophis leonardi* and the other species currently recognized in the genus *Rhabdophis.* See Materials and Methods for the data sources.

Species	DSR	First dorsal scale row	VEN	SC	PRO	PTO	MT^a^
*R. adleri*	19–19‐17	Feebly keeled	150–164	73–88	1–2	3–4	27(25 + 2)
*R. akraios*	19–19‐17	Keeled	167–184	44–66	1	3	17(15 + 2)
*R. angeli*	16(15)‐15–15	Smooth	117–126	39–46	1	3	22–23
*R. auriculatus*	17–17‐15	Strongly keeled	143–162	71–93	1–2	3	27–32
*R. barbouri*	19 at midbody	Strongly keeled	148–156	75–92	2	3	No data
*R. bindi*	19–19‐17	Keeled	157–164	76–102	1	3	26–29 + 2
*R. callichroma*	19 at midbody	Keeled	152–159	79–86	1–2	3	27–35
*R. callistus*	21 at midbody	Strongly keeled	156	76	1	4	No data
*R. ceylonensis*	19 at midbody	Strongly keeled	133–141	48–54	2	3	No data
*R. chrysargoides*	21 at midbody	Strongly keeled	154–161	64–79	1	3	24(22 + 2)
*R. chiwen*	15–15‐15	Smooth	135–162	45–59	1–2	2–3	No data
*R. chrysargos*	19–19‐17	Strongly keeled	143–175	60–93	1–2	3	27–35
*R. confusus*	19–19‐17	Smooth	144–158	56–79	1	3–4	No data
*R. conspicillatus*	19 at midbody	Keeled or smooth	138–147	40–53	1	3	No data
*R. flaviceps*	19 at midbody	Strongly keeled	120–138	49–60	1	3–4	20–22(18–20 + 2)
*R. formosanus* ^b^	19 at midbody	Keeled	163–171	85–88	No data	No data	No data
*R. guangdongensis*	15–15‐15	Smooth	126	39	1	2	20
*R. helleri*	(17,18)19(21)‐19‐(15)17	Smooth	157–178	75–97	1	2–4	21–25(19–23 + 2)
*R. himalayanus*	19–19‐17	Feebly keeled	165–171	82–88	2	3	26(24 + 2)
*R. lateralis* ^b^	19 at midbody	Keeled or smooth	144–188	38–74	1–2	3–4	20–21 + 2
***R. leonardi***	17/18–17/15–15	Smooth	145–159	43–62	1–2	2–3	18–19(16–17 + 2)
*R. lineatus*	19 at midbody	Strongly keeled	132–142	66–71	2	3	18(16 + 2)
*R. murudensis*	19–19‐15(17)	Feebly keeled	176–185	63–97	1	3	23(21 + 2)
*R. nigrocinctus*	19–19‐17	Keeled or smooth	150–170	80–97	1	3–4	28(26 + 2)
*R. nuchalis*	15–15‐15	Smooth	144–169	35–65	1	3	18–22
*R. pentasupralabialis*	15–15‐15	Smooth	135–162	43–64	1	2–3	18
*R. plumbicolor*	23–27 at midbody	Strongly keeled	144–160	35–50	2	3–4	14–15(12–13 + 2)
*R. rhodomelas*	19 at midbody	Strongly keeled	128–138	42–58	1	3–4	14–17(12–15 + 2)
*R. siamensis*	(17,18)19(21)‐19‐(16)17(18)	Smooth	137–156	65–89	1	2–4	22–25(20–23 + 2)
*R. subminiatus*	(17)19(21)‐19–17	Smooth	132–145	59–78	1	3	24–27(22–25 + 2)
*R. swinhonis*	15–15‐15	Weakly keeled or smooth	124–165	44–74	1	2–3	19–23(17–21 + 2–3)
*R. tigrinus*	21–19‐17	keeled	149–170	56–86	2	3	20–25(18–23 + 2)

^a^ Total number of maxillary teeth (the number of maxillary teeth before the diastema + the number of maxillary teeth behind the diastema).

^b^ Morphological characters for *R. formosanus* and *R. lateralis* have not been summarized since those species were partitioned from *R. tigrinus*.

Within China, males of *Rhabdophis leonardi* most closely resemble *R. helleri* and *R. siamensis* in coloration. *R. helleri* was recently elevated from a subspecies of *R. subminiatus* to a full species by David and Vogel (2021), an action supported on molecular evidence by Liu et al. (2021), and the species has a wide range across southern China. *R. siamensis*, also resurrected from the synonymy of *R. subminatus* by David and Vogel (2021), occurs primarily south of China, with its northernmost locality just within southeastern Xishuangbanna Dai Autonomous Prefecture, adjacent to northern Laos. Both sexes of *R. helleri* and *R. siamensis* resemble male *R. leonardi* in having a green dorsal ground color and a broad region of red across the neck, although the red region tends to extend farther posteriorly in *R. helleri* and *R. siamensis*. A third population, *R. confusus* on Hainan Island, also was split from *R. subminiatus* by David and Vogel (2021), but that species lacks a distinct red nuchal region, with only scattered areas of red on an otherwise green dorsum. *R. helleri*, *R. siamensis*, and *R. confusus* all have 19 dorsal scale rows at midbody (versus 15–17 in *R. leonardi*) and reach larger adult sizes than *R. leonardi* (to approximately 800 mm total length in *R. siamensis* and *R. confusus*, and up to 1300 mm in *R. helleri*, versus about 670 mm in *R. leonardi*) (David & Vogel, 2021; Zhao, 2006). For the remaining congeners, both in China and across the range of the genus, *R. leonardi* can be readily distinguished by its DSR, VEN, SC, PRO, PTO, MT. A comparison of all currently recognized species of *Rhabdophis* is provided in Table 3.

#### 3.5.5 Habitat, behavior, and distribution

**FIGURE 8:**
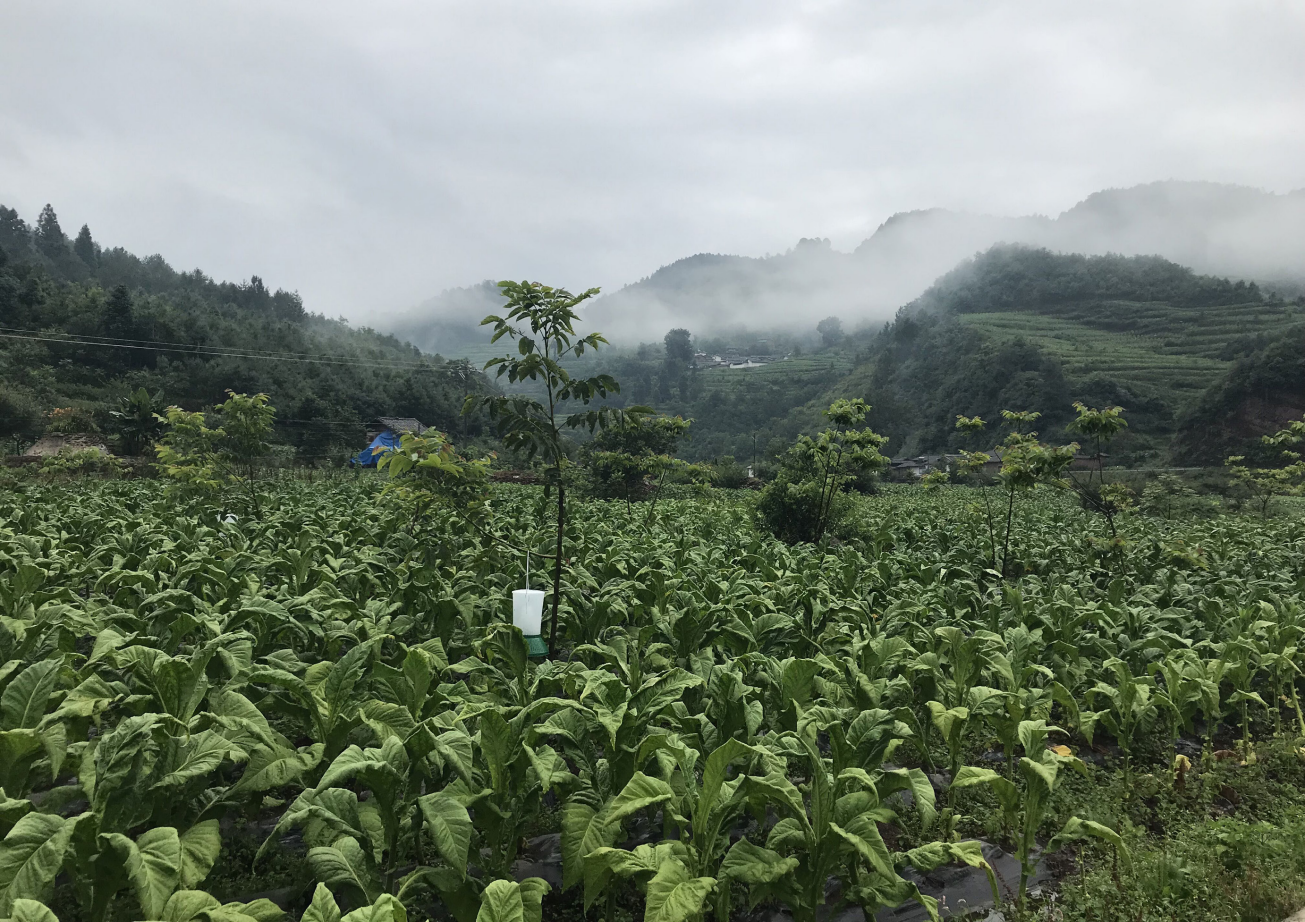
Habitat of *Rhabdophis leonardi* (site of capture of SICAU201705031) in Xiaoshanbao village, Huili County, Liangshan Yi Autonomous Prefecture, southern Sichuan Province, China. (photo by Shijun Yang).

Residents of Xiaoshanbao Village (Figure 8) report that they occasionally encounter this species. Therefore, we suggest that this species occurs in southern Sichuan and adjacent Yunnan at approximately 1730–2230 m elevation, although it occurs at higher elevations at more northern localities.

A blurry photograph of a toad being consumed by this species (Figure 5d) also was obtained from a resident of Huangcao Village. *R. leonardi*, therefore, is known to feed on earthworms and slugs (Yoshida et al., 2020; Zhao, 2006; Zhao et al., 1998), as well as anuran amphibians. We also note a catalog entry at the California Academy of Science indicating that CAS 215027, identified in the catalog as *R. leonardi*, contained snails in its stomach.

Like other *Rhabdophis*, *R. leonardi* is oviparous. Four eggs were laid by SICAU201705027, one of which was incubated in the laboratory, where it hatched after 49 days at 25°C.

In addition to the vouchered specimens described above, one of us (LD) observed this species in the mountains west of Kunming, Yunnan Province, China, and documented the individual photographically (Figure 5e,f), although it was not collected nor were measurements recorded.

We also note here the presence of eight specimens at the California Academy of Science (CAS) that are identified as *Rhabdophis leonardi*, based on catalog data returned by a search of the species name on VertNet (http://vertnet.org; accessed 2 April 2022). Seven of those specimens are from Yunnan Province, China, six of which are from Nu Jiang Nature Reserve, on the western slope of Gaoligongshan (25.994444° N, 98.658056° E), at approximately 2475 m elevation. One of those six, CAS 215027, was sequenced by Deepak et al. (2022) and included in their phylogeny. Specimens from that site would appear to be Clade C, based on the proximity of the locality to the southernmost genotype of Clade C reported by Zhu et al. (2022). A seventh CAS specimen identified as *R. leonardi* (CAS 234494), a male, is from nearby in Nujiang Prefecture (26.001667° N, 98.661111°E). Of the seven CAS specimens from China cataloged as *R. leonardi*, five are sexed, three males and two females. An eighth specimen at the CAS is from Chipwe Township, Myanmar, NE of Myitkyina (25.709444^o^N, 98.329444° E;1897 m elevation), in Kachin State. That site lies well north of the type locality of *R. leonardi* and is also closer to known localities for Clade C.

Interestingly, a female specimen at the CAS that is cataloged as *R. nuchalis* (CAS 234408), from Nujiang Prefecture (28.129167° N, 98.831944° E), appears in the phylogeny of Deepak et al. (2022) as sister to CAS 215027, from which it is minimally differentiated. That raises the question of whether CAS 234408 may be a misidentified specimen of *R. leonardi*, an error that appears more likely given the nuchal pattern of females of the latter species.

## 4 DISCUSSION

Species of the genus *Rhabdophis* are widely distributed across southern and eastern Asia, with an elevational range from sea level to more than 3000 m (Zhao, 2006). The existence of cryptic species of *Rhabdophis* has been suspected (Liu et al., 2021; Takeuchi et al., 2018), and several of those have recently been described (David & Vogel, 2021; Zhu et al., 2022). *R. leonardi*, as currently recognized, has a wide distribution in southwestern China and adjacent Myanmar. However, our data and those of Zhu et al. (2022) suggest that the two well‐defined molecular lineages of nominal *R. leonardi*, referred to here as Clades B and C of Zhu et al. (2022), may be distinct at the specific level. Clade B included specimens from northern, central, and west‐central Yunnan Province, as well as extreme southwestern Sichuan Province. The nine specimens of *R. leonardi* discussed here, eight from Sichuan Province and one from Dali, Yunnan Province, were included in Zhu et al. (2022) and were found to belong to Clade B. In contrast, Clade C included samples from far northwestern Yunnan Province, close to the border with Myanmar, and from the adjacent Xizang (Tibet) Autonomous Region. Furthermore, the southernmost sample in Clade C and the westernmost sample from Clade B in Zhu et al. (2022) were located very close to each other, approximately 70 km apart, with no obvious geographic barrier between them. Although that point of virtual parapatry between Clades B and C is relatively close to the type locality of *R. leonardi*, the type locality nonetheless is closer geographically to known localities of Clade B. Thus, the relationship between the type locality of *R. leonardi* and the known distribution of the two genotypes of nominal *R. leonardi* makes it likely that the holotype belongs to the more southeastern population, Clade B. That said, the complex topography of the border region where these populations occur suggests that caution should be exercised in making assumptions about the genotype(s) present at any locality for which sequences are unavailable.

Males of *R. leonardi* superficially resemble *R. helleri* in coloration, and local people usually considered that male *R. leonardi* at relatively high elevation sites represented individuals of *R. helleri*. (Below about 1700 m, individuals regarded as *R. helleri* appear to have been correctly identified.) It is possible that male *R. leonardi* are Batesian mimics of *R. helleri*, which is a member of the dangerously venomous *R. subminiata* complex. Given that resemblance, males of *R. leonardi* may well have been misidentified in the past, and specimens of both species residing in collections should be carefully re‐examined. Importantly, Mulcahy et al. (2022) recently emphasized the importance of verifying the identifications of voucher specimens for tissue samples and of updating the identifications in open databases. We have noted such doubts above with respect to the identification of specimens in the complex *R. nuchalis* Group.

*Rhabdophis leonardi* appears to prey in part upon anuran amphibians, including bufonids, in contrast to at least some of the other, and generally smaller, members of the earthworm‐eating *R. nuchalis* Group to which *R. leonardi* belongs (Piao et al., 2020; Yoshida et al., 2020). For example, *R. pentasupralabialis* has a total length of approximately 483 mm and *R. chiwen* has a total length of about 536 mm (Piao et al., 2020), versus about 600–700 mm in *R. leonardi*. In all these aspects of diet and size, *R. leonardi* appears intermediate between other members of the *R. nuchalis* Group and more generalized species of *Rhabdophis*. Diet may also be reflected in the lower number of anterior dorsal scale rows in *R. chiwen*, *R. nuchalis*, and *R. pentasupralabialis* (with 15‐15‐15 rows) compared to *R. leonardi* (17–17/15–15 rows). A higher number of dorsal scale rows corresponds to more regions of expansible skin between the scales. Thus, the higher number of anterior dorsal rows may function in accommodating larger prey anterior to the pylorus.

Finally, our molecular results confirm previous findings that earlier concepts of *Rhabdophis* were not monophyletic. Specifically, *R. conspicillatus* belongs to *Xenochrophis*, as revealed by other recent studies (Deepak et al., 2022; Purkayastha et al., 2018; Takeuchi et al., 2018).

## AUTHOR CONTRIBUTIONS

**Shijun Yang:** Conceptualization (equal); data curation (equal); formal analysis (equal); investigation (equal); methodology (equal); software (equal); writing – original draft (equal); writing – review and editing (equal). **Alan H. Savitzky:** Conceptualization (equal); data curation (equal); investigation (equal); methodology (equal); validation (equal); writing – original draft (equal); writing – review and editing (equal). **David J. Gower:** Investigation (equal); methodology (equal); writing – review and editing (equal). **V Deepak:** Investigation (equal); methodology (equal); writing – review and editing (equal). **Akira Mori:** Investigation (equal); methodology (equal); writing – review and editing (equal). **Khot Rahul:** Investigation (equal); methodology (equal); writing – review and editing (equal). **Jingsong Shi:** Investigation (equal); methodology (equal); writing – review and editing (equal). **Li Ding:** Investigation (equal); methodology (equal); writing – review and editing (equal). **Mian Hou:** Investigation (equal); methodology (equal); writing – review and editing (equal). **Haiyuan Xu:** Investigation (equal). **Qin Wang:** Investigation (equal); writing – review and editing (equal). **Guangxiang Zhu:** Conceptualization (equal); data curation (equal); funding acquisition (equal); resources (equal); supervision (equal); writing – review and editing (equal).

## CONFLICT OF INTEREST STATEMENT

The authors declare no conflicts of interest.

## Data Availability

Data sharing is not applicable to this article as no datasets were generated or analyzed during the current study.

## APPENDIX 1

### SPECIMENS OF *RHABDOPHIS LEONARDI* EXAMINED. CLADE DESIGNATIONS CORRESPOND TO THOSE OF ZHU ET AL. (2022)

Holotype of *Natrix leonardi* Smith 1923: **BMNH** 1946.1.12.86 from Sinlum Kaba, Kachin State, Myanmar.

*Rhabdophis leonardi*, Clade B (5): **SICAU** 201705026 and **SICAU** 201705031 from Huili, Sichuan, China; **SICAU**201807001, **SICAU**201705027, and **SICAU** 20170726 from Panzhihua, Sichuan, China.

*Rhabdophis leonardi*, Clade C (10): **KIZ** 791327 and **KIZ** 791337 from Yongde Snow Mountain; **KIZ** 791324, **KIZ** 791370, **KIZ** 791381‐791382, **KIZ** 791389 from Manlai, Yongde County; **RDQ** 2016000063 from Lushui County; **RDQ** 2014001366 from Daliang Moutain, Yun County; **RDQ** 2014001399 from Linxiang District, Lincang County.
